# Drug Repurposing Opportunities in Pancreatic Ductal Adenocarcinoma

**DOI:** 10.3390/ph14030280

**Published:** 2021-03-20

**Authors:** Rita Rebelo, Bárbara Polónia, Lúcio Lara Santos, M. Helena Vasconcelos, Cristina P. R. Xavier

**Affiliations:** 1 Cancer Drug Resistance Group, IPATIMUP—Institute of Molecular Pathology and Immunology, University of Porto, 4200-135 Porto, Portugal; rrebelo@ipatimup.pt (R.R.); bpolonia@i3S.up.pt (B.P.); 2 i3S—Instituto de Investigação e Inovação em Saúde, Universidade do Porto, 4200-135 Porto, Portugal; 3 Experimental Pathology and Therapeutics Group, IPO—Instituto Português de Oncologia, 4200-072 Porto, Portugal; llarasantos@gmail.com; 4 ICBAS—Biomedical Sciences Institute Abel Salazar, Universidade do Porto, 4050-313 Porto, Portugal; 5 Department of Biological Sciences, FFUP—Faculty of Pharmacy of the University of Porto, 4200-135 Porto, Portugal

**Keywords:** drug repurposing, pharmacology, pancreatic cancer, therapeutic strategies

## Abstract

Pancreatic ductal adenocarcinoma (PDAC) is considered one of the deadliest tumors worldwide. The diagnosis is often possible only in the latter stages of the disease, with patients already presenting an advanced or metastatic tumor. It is also one of the cancers with poorest prognosis, presenting a five-year survival rate of around 5%. Treatment of PDAC is still a major challenge, with cytotoxic chemotherapy remaining the basis of systemic therapy. However, no major advances have been made recently, and therapeutic options are limited and highly toxic. Thus, novel therapeutic options are urgently needed. Drug repurposing is a strategy for the development of novel treatments using approved or investigational drugs outside the scope of the original clinical indication. Since repurposed drugs have already completed several stages of the drug development process, a broad range of data is already available. Thus, when compared with *de novo* drug development, drug repurposing is time-efficient, inexpensive and has less risk of failure in future clinical trials. Several repurposing candidates have been investigated in the past years for the treatment of PDAC, as single agents or in combination with conventional chemotherapy. This review gives an overview of the main drugs that have been investigated as repurposing candidates, for the potential treatment of PDAC, in preclinical studies and clinical trials.

## 1. Drug Repurposing: An Attractive and Challenging Approach

Drug repurposing has been emerging as an interesting alternative to the conventional drug discovery process. Additionally referred to as “drug repositioning”, “drug reprofiling”, “drug redirecting”, “drug rediscovery” or “drug re-tasking”, this is a strategy for the development of novel treatments using approved or investigational drugs outside the scope of the original clinical indication [1,2,3]. Interestingly, the recent development and increasing interest observed in genomic and proteomic technologies for the assessment of cancer specific biological pathways has led to the discovery of several new drug targets, providing excellent opportunities for drug repurposing, since almost every drug used in human therapy has the potential to address more than one target [3]. The increasing interest in this approach relies on the fact that, by finding new applications for clinically approved drugs, drug repurposing is able to overcome the major issues associated with conventional drug discovery, which include substantial costs, slow pace and high risk of failure [3,4]. According to Nosengo (2016), the estimated cost for a repurposed drug to reach the market is US$300 million, whereas, for a new drug, it is estimated to be ~$2 to $3 billion [5]. Interestingly, the Food and Drug Administration (FDA) approval for new drugs has decreased in the past years, especially when compared to the 1990s [6].

**Figure 1:**
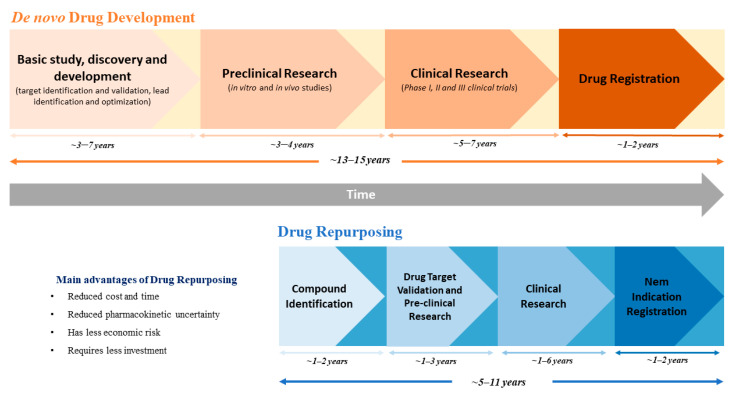
Schematic comparison between the processes and timeline of *de novo* drug development and drug repurposing.

In fact, clinically approved drugs have previously completed several stages of the drug development process; hence, data regarding dosing, safety, toxicity, drug interactions, side effects, pharmacodynamics and pharmacokinetics is often available [1,2,3]. Therefore, the need for large investments and the time frame for drug development can be significantly reduced [1,3]. As depicted in Figure 1, while new drug discovery and development might take up to 10 to 17 years for a drug to reach the market, the timeline for drug repurposing is only about 3 to 12 years [4]. Additionally, the previous evidence on the effect of these drugs in preclinical models and in humans reduces the risk of failure in upcoming clinical trials [1]. Nevertheless, despite its great advantages, drug repurposing can face several obstacles, particularly in drug licensing, clinical adoption or even during the development of clinical trials [1,2]. These problems might be explained by the existence of regulatory exclusivities, which increase drug market protection beyond the patent term, or by drug withdrawal from the market, which hampers drug supply for clinical trials, as well as by complications in the label extension in medicines that are authorized through national procedures [1,2].

Nonetheless, drug repurposing represents a relatively unexplored source of new therapies, being a very attractive option, especially in neglected areas of medicine, where the lack of commercial interest delays drug development. Moreover, drug repurposing candidates that are off-patent offer an additional advantage. The competitive environment in the pharmaceutical industry leads to a severe markdown in their prices, which facilitates access to medicines for the general population and reduces the public health burden [2].

Initially, drug repurposing was largely a serendipitous process or was based on clinical side effects and off-label use [1,3]. One of the first cases of drug repurposing was thalidomide, a sedative and antiemetic drug that was found to be effective in the treatment of erythema nodosum leprosum and, later, in multiple myeloma. This led to the development of analogs, such as lenalidomide and pomalidomide, which are nowadays successfully used in the treatment of multiple myeloma [1]. Other popular cases resulted from different repurposing approaches, such as a retrospective clinical analysis or pharmacological analysis [1,3]. Most recently, systematic approaches have been employed to identify novel candidates for drug repurposing, namely computational (molecular docking, pathway mapping, genetic association, signature matching, etc.) and experimental ones (binding assays and phenotypic screening) [1]. 

Despite the great advances in technology and the growing knowledge of cancer, the oncological field still faces serious challenges, including the limited success of current therapies and the decline in new oncology drugs approval, along with the increasing incidence of cancer and the lack of treatments for rare cancers [6,7,8,9]. Therefore, drug repurposing could respond to some of the existing unmet needs in this field. In fact, several drugs have already been successfully repurposed in the cancer field. Some examples are listed in Table 1. 

For instance, the chemotherapeutic drug gemcitabine was initially designed as an antiviral drug but, afterwards, was found to have antitumor activity in vivo [10]. Nowadays, gemcitabine is used to treat a wide variety of cancers, being the gold standard chemotherapeutic drug in pancreatic cancer [11]. 

**Table 1:** Successful examples of drug repurposing in cancer treatment.

Drug	Original Indication	New Clinical Indication in Cancer Treatment	Date of Approval	Mechanism of Action	References
Retinoic acid/Tretinoin	Acne	Acute promyelocytic leukemia	1996	Binds to retinoic acid receptors and degrades the fusion protein PML-RARa	[12]
Interferon α2b	Hepatitis B and C	Melanoma, Multiple myeloma, Hairy cell leukemia, Carcinoid tumor, Follicular lymphoma	2000	Immunomodulatory activity	[13]
Thalidomide	Sedative/anti-emetic	Multiple myeloma	2006	Immunomodulatory, anti-inflammatory and potential anti-neoplastic activities due to inhibition of tumor necrosis factor-alpha (TNF-α) production	[14]
Raloxifene	Osteoporosis	Breast cancer	2007	Selective estrogen receptor modulator (SERM) that acts like an antagonist in uterine and breast tissues	[15]
Gemcitabine	Antiviral	Bladder cancer; Pancreatic ductal adenocarcinoma; Non-small cell lung cancer; Ovarian cancer; Breast cancer	2009	Pyrimidine antimetabolite inhibits DNA synthesis, leading to apoptosis and arresting tumor growth	[16]
Pomalidomide	Thalidomide derivative	Multiple myeloma	2013	Cytotoxic and immunomodulatory effects. Suppresses angiogenesis by blocking the migration and adhesion of endothelial cells	[17]
Itraconazole	Antifungal	Nevoid basal-cell carcinoma (Gorlin syndrome)	2017	Binds to Smoothened (SMO) protein, blocking the Hedgehog signaling pathway and limiting the growth and spread of tumoral cells	[18]
Lenalidomide	Thalidomide derivative	Multiple myeloma	2018	Cytotoxic and immunomodulatory effects due to the degradation of lymphoid transcriptional factors	[19]
Arsenic	Tuberculosis and syphilis	Acute promyelocytic leukemia	2020	Causes fragmentation of DNA and degrades the fusion protein PML-RARa	[20]

As highlighted in the next section, pancreatic cancer is a very challenging disease. The difficult early diagnosis and lack of effective therapies creates an urgency for new therapeutic options to treat this high-mortality malignancy. Therefore, drug repurposing emerges as a resourceful strategy to accelerate the discovery of novel and effective treatments, with a rapid clinical translation. This review focuses on the current panorama of pancreatic cancer and summarizes the most recent studies on potential drug repurposing candidates for the treatment of this disease. 

## 2. Pancreatic Cancer: The Current Picture

According to Global Cancer Observatory, in 2018, pancreatic cancer was considered the seventh deadliest cancer worldwide, despite being only the 12th with most incidence, and its mortality is estimated to increase up to ~80% by 2040 [21]. With a five-year survival rate of approximately 5%, pancreatic cancer is one of the cancers with the poorest prognosis, and no major advances in cancer treatments have been done in the past 20 years [11,22]. Mortality and incidence rates have not improved in the last decades [23]. Therefore, new strategies of drug development need to be implemented. 

Pancreatic cancer affects mainly the elderly population [24]. The vast majority of patients are diagnosed at late stages, already presenting a metastatic or locally advance disease, which hampers surgical excision [11,25]. Only around 10% of pancreatic carcinomas are caused by inherited germlines mutations, the majority of cases due to nongenetic risk factors, such as chronic pancreatitis and diabetes (nonmodifiable factors), smoking, obesity, diet and alcohol abuse (modifiable factors) [11,26]. Infections caused by *Helicobacter pylori*, hepatitis B and human immunodeficiency virus also appear to increase the relative risk of pancreatic cancer [11].

Pancreatic cancer can develop from both exocrine and endocrine cells; however, exocrine tumors represent approximately 95% of all cases (with only 2% of these being benign), while the remaining 5% corresponds to endocrine tumors, often known as Pancreatic Neuroendocrine Tumors (pNETs) [11]. The most common type is Pancreatic Ductal Adenocarcinoma (PDAC), which accounts for approximately 85% of all pancreatic cancers; thus, it is usually referred to as “pancreatic cancer” [11,26,27,28,29,30]. For this reason, our review will focus on this type of pancreatic cancer.

PDAC follows the adenoma-to-carcinoma model, presenting a stepwise evolution from noninvasive preneoplastic lesions to invasive tumors [11,24,26,30,31,32]. Intraepithelial neoplasms (PanINs) are the most frequent precursor lesions and commonly harbor mutations in *KRAS*, *CDKN2A*, *TP53* and *SMAD4* genes [33,34,35,36]. However, recent whole-genome sequencing studies are challenging this model, suggesting that the simultaneous impairment of these genes might occur in some cases, possibly through chromothripsis, rather than the sequential accumulation of mutations by precursor lesions [37]. Nonetheless, it is well-known that more than 90% of pancreatic cancers harbor a *KRAS* mutation and that silencing mutations in tumor-suppressor genes, especially in *CDKN2A* (80–95%), *TP53* (50–70%) and *SMAD4* (>50%), can simultaneously occur [11,26,38,39]. Additionally, somatic aberrations in DNA mismatch repair genes, like *MLH1* and *MSH2*, may also be found [11]. 

As aforementioned, PDAC remains a very difficult disease to treat, and several factors can be accountable for this failure. First, early detection is extremely difficult, since PDAC is often asymptomatic until reaching an advanced or metastatic sate [26]. The second issue is the lack of an accurate and inexpensive screening method for the general population [26]. Unfortunately, surgical resection is only possible in 15–20% of patients, and the five-year survival rate is approximately 20% [11,40]. In addition, the local and systemic recurrence rates after resection are relatively high, reaching up to 60% and 90% of cases, respectively [38,41]. Unfortunately, the majority of PDAC patients are not even eligible for surgery, and cytotoxic chemotherapy remains the basis of systemic therapy. In patients with borderline resectable tumors, chemotherapy is used as a neoadjuvant therapy, and in patients with locally advanced or metastatic tumors, it is used to reduce disease burden or as a palliative treatment, respectively [38,41]. However, no major advances have been made regarding PDAC treatment; thus, few therapeutic options are available, and these are highly toxic to patients. The most frequently used drugs in chemotherapy regimens for PDAC treatment include gemcitabine, FOLFIRINOX (leucovorin/5-fluorouracil/irinotecan/oxaliplatin), nab-paclitaxel, erlotinib, capecitabine, GTX (gemcitabine/docetaxel/capecitabine) and irinotecan [42]. In addition, the combinatory treatment of gemcitabine with cisplatin is used in tumors with *BRCA1/2* mutations, whereas pembrolizumab is used as a second-line therapy in tumors with Microsatellite Instability-High or deficient Mismatch Repair [42]. 

In the last decade, there were two major improvements in the current standard care of metastatic PDAC. First, in 2013, the combination of gemcitabine and nab-paclitaxel was approved for the first-line treatment of patients with metastatic PDAC, following the report from Von Hoff et al. showing that this combination could improve the overall survival, progression-free survival and response rate compared to gemcitabine alone, although it presented an increased toxicity [11,43,44]. More recently, in 2016, a phase III trial conducted by Wang-Gilam et al. demonstrated that nanoliposomal irinotecan could extend the overall survival in combination with 5-fluorouracil (5-FU)/leucovorin in patients with metastatic PDAC who progressed after gemcitabine treatment, with a manageable safety profile [45]. Therefore, the FDA and European Medicines Agency (EMA) have approved this new treatment as a second-line regimen [46]. 

Finally, another obstacle in pancreatic cancer treatment is its unique tumor microenvironment (TME), which is composed of extracellular matrix, cancer cells and stromal cells, including pancreatic stellate cells (PSCs), fibroblasts, myeloid-derived suppressor cells (MDSCs), regulatory T cells (Tregs) and tumor-associated macrophages (TAMs) [47,48]. When activated by cancer cells, PSCs produce stromal components, such as laminin, fibronectin and collagens, a process known as desmoplasia that is found both in primary and metastatic PDAC tumors [47,48,49,50,51,52]. Studies show that, although stroma’s primary function would be to restrain tumor growth, angiogenesis and metastization, there is a reprogramming of stromal cells by cancer cells to support tumorigenesis [48,53,54]. This results in an intense stromal reaction that acts like a mechanical barrier around the tumor and hampers the appropriate vascularization, limiting immune cell infiltration and drug delivery [11,55]. Given the complexity of the stroma in PDAC and the high heterogeneity of the cellular populations that comprised it, stroma-targeted therapies might exert divergent effects; therefore, this type of therapy should be carefully evaluated in this type of cancer [48,56].

## 3. Drug Repurposing Candidates for Pancreatic Cancer Treatment

In the past years, several studies have been investigating the potential of drug repurposing candidates for the treatment of pancreatic cancer, as single agents or in combination with conventional chemotherapy. These candidates target multiple hallmarks of cancer (replicative immortality, resistance to cell death, deregulated metabolism, sustained angiogenesis and evasion to the immune system, tissue invasion and metastasis,) and belong to very different pharmacological classes, from antibiotics to antipsychotics. 

As summarized in Table 2, this section highlights several preclinical studies that have been evaluating the potential of selected drug repurposing candidates for the treatment of PDAC, either targeting pancreatic cancer cells or cells from the TME. Nonetheless, many of these candidates still need further studies to confirm their anticancer efficacy.

### 3.1. Preclinical Studies

#### 3.1.1. Carglumic Acid

This orphan drug is commonly used to treat hyperammonemia, both in adult and pediatric patients. Surprisingly, a study demonstrated that carglumic acid is able to suppress 50% of cell viability in human and murine PDAC cell lines through induction of apoptosis [57]. Interestingly, the authors demonstrated that, while gemcitabine was more toxic to a human pancreatic ductal epithelial cell line than to PDAC cells, carglumic acid was more effective at inhibiting cancer cells than normal cells proliferation [57]. Additionally, carglumic acid showed promising in vivo effects by reducing 80% of the tumor growth in an orthotopic PDAC model [57].

#### 3.1.2. Warfarin

In vitro and in vivo studies conducted by Kirane et al. (2015) pinpointed warfarin, an anticoagulant and vitamin K antagonist, as a very promising drug repurposing candidate in Axl-expressing pancreatic tumors [58]. This study showed that, by inhibiting Gas6 (a vitamin K-dependent ligand of the receptor tyrosine kinase Axl), low-dose warfarin impairs tumor cell growth, migration, invasiveness, angiogenesis and metastasis, while increasing the expression of apoptotic markers, without any complications in coagulation [58]. Interestingly, the same research group also demonstrated that low-dose warfarin could sensitize PDAC tumor cells to gemcitabine and nab-paclitaxel therapy in a KIC (*p48^Cre^*; *LSL-Kras^G12D^*; *Cdkn2a^f/f^*) mouse model [59]. 

An immunomodulatory effect has also been described for low-dose warfarin in PDAC without any effect in coagulation [60]. Recently, Tormoen et al. observed that warfarin suppresses tyrosine-protein kinase Mer (MerTK) signaling, enhancing an adaptive immune response after Stereotactic Ablative Radiotherapy (SABR). MerTK is mainly expressed in macrophages and regulates the phagocytosis of apoptotic cancer cells, eliminating tumor antigens that could trigger an antitumor immune response and allowing irradiated tumor cells to evade immune control. Therefore, patients with PDAC undergoing SABR could benefit from warfarin as a stimulator of an antitumor adaptive immune response [60]. Regarding clinical evidence, a retrospective study found that a treatment with low-dose warfarin improved patient survival, independently of the type of chemotherapy received [61].

#### 3.1.3. Metformin

A well-known antidiabetic drug, approved for type 2 diabetes mellitus (T2DM), is metformin, an oral antihyperglycemic drug [94]. As aforementioned, diabetes is a risk factor for PDAC, and it has been associated with a worse patient’s outcome [95,96]. Interestingly, retrospective studies have shown that diabetic patients with pancreatic cancer (or other solid tumors) that were treated with metformin presented a cancer survival benefit when compared with patients treated with other antidiabetic drugs (for example, insulin or sulfonylureas [97]. These findings may be related to metformin’s ability to influence various cellular pathways, including the activation of the LKB1/AMPK pathway, inhibition of cell division, promotion of apoptosis and autophagy and activation of the immune system [98]. Indeed, numerous studies have investigated the antitumor potential of metformin in a variety of cancers, alone or in combination with conventional chemotherapy. Besides its antidiabetic activity, metformin also exhibits anticancer effects, targeting multiple hallmarks of cancer both in vitro and in vivo. This anticancer effect has been explained by inhibition of mTOR, which prevents protein synthesis and cell growth, activation of the AMPK pathway, triggering activation of the tumor suppressors TSC2 and P53 and MAPK regulation, which controls cell proliferation, differentiation and survival, among others [99]. 

Candido et al. (2018) demonstrated that, although metformin could not inhibit cell growth by itself, a suboptimal dose of this drug was able to potentiate the effect of gemcitabine, 5-FU and cisplatin in different PDAC cell lines [62]. In fact, the association of metformin with conventional chemotherapy to treat PDAC is under evaluation in several clinical trials, as described in Section 3. Interestingly, the studies conducted by Chen et al. (2017) concluded that metformin also suppressed cancer initiation. This drug prevented the formation of precursor lesions and suppressed chronic pancreatitis-induced tumorigenesis in KC (LSL-KrasG12D; Pdx1-Cre) mice, partly by inhibiting cell proliferation and blocking STAT3 signaling [63]. Moreover, metformin improved the overall survival, reduced tumor volume and decreased abdominal invasions in KPC (LSL-Kras^G12D/+^; LSL-Trp53^R172H/+^; Pdx1-Cre) mice, also playing a positive effect in pancreatic desmoplasia [63]. Furthermore, the activation of AMPK signaling and downregulation of p-mTOR were also observed in mice treated with metformin [63]. Additionally, the in vitro and in vivo results demonstrated that metformin could inhibit cell migration and invasion, as well as revert the expression of Epithelial-to-Mesenchymal Transition (EMT) markers, possibly through the suppression of TGF-β1/Smad2/3 signaling [64]. 

Evidence regarding whether pancreatic cancer patients might benefit from treatment with metformin is often contradictory. For instance, a retrospective study found that patients with T2DM and pancreatic cancer taking metformin had an increased overall survival when compared with patients in the same situation that were not taking metformin [100]. Contrary to these results, a randomized phase 2 trial in which 121 patients with advanced PDAC were randomly assigned to receive gemcitabine and erlotinib with either placebo (n = 61) or metformin in a conventional antidiabetic dosage (n = 60) showed no improvement in the clinical outcomes of patients belonging to the metformin group. It is important to note that this study included nondiabetic patients in both arms (87% in the placebo group and 90% in the one receiving metformin), and patients with previous treatment with metformin or erlotinib within six months before the beginning of the study were not enrolled [101]. A meta-analysis performed by Dong et al. (2017) concluded that, while cohort studies favored the use of metformin in patients with pancreatic cancer, randomized controlled trials did not [102]. The discrepancies observed among studies might arise from several factors. Some observational studies overestimate the effects of metformin by using a time-fixed analysis, while others only include pancreatic cancer patients with diabetes who can benefit from metformin due to the concurrent disease. Besides, some studies do not differentiate pancreatic cancer stages, making it difficult to draw conclusions.

Interestingly, emerging evidence suggests a novel mechanism for the positive effect of metformin in PDAC patients. Several studies have demonstrated that microbiome plays a role in oncogenesis and can modulate cancer treatment response in several cancer types, including PDAC [103,104,105,106,107,108,109]. Moreover, several studies have reported that metformin modulates the gut microbiota in T2DM models, suggesting that its antidiabetic effect is also microbial-dependent [110,111,112]. Recently, Dong et al. (2019) showed that alterations in the duodenal microbiome composition, i.e., increased levels of *Clostridium sensu stricto*, were linked to PDAC development in KC mice undergoing a high fat and calories diet (HFCD) and that the treatment with metformin could decrease PDAC formation by reverting the *Clostridium sensu stricto* levels [113].

#### 3.1.4. Monensin

This veterinary antibiotic is the product of natural fermentation by *Streptomyces cinnamonensis* and presents iontophoretic activity, forming a complex with monovalent cations and disrupting cell homeostasis, which leads to cell death [114]. Given its toxicity, monensin is not used in humans; however, it has been studied for drug repurposing in different types of malignancies, including pancreatic cancer [4]. Wang et al. (2018) demonstrated that monensin suppressed the cell proliferation and migration of gemcitabine resistant PDAC cells through apoptosis and cell cycle arrest at G1 and showed a synergistic effect with gemcitabine and erlotinib in suppressing cell growth and in inducing cell death [65]. Mechanistically, these authors demonstrated that monensin blocked the E2F/DP1, signal transducer and activator of transcription 1/2 (STAT1/2), nuclear factor-κB (NF-Kb), activator protein 1 (AP-1) and ETS Like-1 protein Elk-1 (Elk-1)/Serum response factor (SRF)pathways and suppressed epidermal growth factor receptor (EGFR) expression [65]. In PDAC xenograft mouse models, monensin impaired tumor growth via the EGFR pathway [65].

#### 3.1.5. Nelfinavir and Nitroxoline 

The HIV protease inhibitor nelfinavir and the antibiotic nitroxoline have been studied for the treatment of pancreatic cancer as single agents or in combination with other drugs. Nelfinavir can sensitize pancreatic tumor cells to radiotherapy and has been tested for the treatment of different stages of pancreatic cancer [115,116,117]. Regarding nitroxoline, this drug has shown antitumor effects in distinct types of cancer, including the suppression of angiogenesis and inhibition of tumor cell migration and invasion [118]. 

Veschi et al. (2018) demonstrated that, while in monotherapy, nelfinavir and nitroxoline reduced cell viability and affected the cell cycle in several PDAC cell lines [66]. When combined with erlotinib, nelfinavir and nitroxoline, it displayed synergistic effects, strongly affecting cell viability through cell cycle arrest and apoptosis [66]. Interestingly, using a shotgun proteomic approach, the same authors demonstrated in another study that the anticancer effects of nitroxoline in PDAC cells were associated with increased reactive oxygen species (ROS) production and induction of the DNA damage response through downregulation of the Na/K-ATPase pump and β-catenin, as well as mitochondrial depolarization and deregulation of cytosolic iron homeostasis [67]. 

#### 3.1.6. Azithromycin, Doxycycline, Tigecycline and Pyrvinium

Taking into consideration the Endosymbiotic Theory, which defends that mitochondria evolved from bacteria, it is no surprising that several classes of antibiotics, including tetracyclines, might have an off-target effect in the mitochondrial function [69,119]. In fact, the tetracycline and chloramphenicol families may inhibit mitochondrial and bacterial translation and affect some complexes of the mitochondrial respiratory chain [120]. Son et al. (2009) reported that doxycycline caused an inhibition of PDAC cell growth, an induction of apoptosis and cell cycle arrest [68]. Moreover, doxycycline reduced 80% of the tumor growth in a PDAC nude xenograft mouse model [68]. Interestingly, Lamb et al. (2015) studied several classes of antibiotics in pancreatic Cancer Stem Cells (CSCs) and showed that those drugs inhibited tumor-sphere formations in vitro and did not present toxicity in noncancer cells [69].

#### 3.1.7. Ritonavir

Another promising antiviral drug that is described to have anticancer activity in both liquid and solids tumors is ritonavir, a FDA-approved drug for HIV treatment [121]. This drug impaired cancer cell growth and showed a synergistic effect with gemcitabine in different PDAC cell lines through alterations in apoptosis, cell cycle and cell motility and invasiveness [70]. The underlying mechanisms of the antitumor effect of ritonavir in vitro appears to be through suppression of the AKT pathway and sequestration of the E2F transcription factor 1 (E2F-1), preventing cell cycle progression to the S phase [70]. In contrast to these results, O’Donoghue et al. (2017) assessed the effect of ritonavir as a molecular inhibitor of cathepsin E (reported to be overexpressed in pancreatic cancer) on the tumor burden of PDAC mice. Although mouse PDAC tumors presented high levels of cathepsin E, ritonavir did not reduce the tumor burden in vivo [71]. However, no other effects of ritonavir in cancer cells were investigated in this study. Therefore, further studies need to be carried out to clarify the potential of this drug in PDAC treatment.

#### 3.1.8. Itraconazole

This broad-spectrum antifungal has been extensively studied as a drug-repurposing candidate in numerous types of cancers [4]. In fact, itraconazole was approved for treatment of nevoid basal-cell carcinoma syndrome (Gorlin syndrome) in 2017, due to its ability to block the Hedgehog signaling pathway by targeting Smoothened [18]. Similar to other malignancies, the Hedgehog signaling pathway is frequently altered in PDAC and was already associated with chemoresistance in this type of cancer [122,123,124]. Interestingly, itraconazole is also reported to inhibit P-glycoprotein, a key player in multidrug resistance in cancer [125,126].

Cheng et al. (2018) reported that itraconazole could inhibit the cell viability, invasion and migration, as well as induce apoptosis, in several PDAC cell lines [72]. This antifungal drug also presented an anti-EMT effect, partly by suppressing TGF-β/SMAD2/3 signaling, which seems essential for its anticancer activity in vitro, and inhibited tumor growth in a KPC mouse model [72]. Consistently, Jiang et al. (2018) also observed that itraconazole reduced the in vitro cell proliferation and tumor growth in a PDAC xenograft mouse model [73]. Moreover, itraconazole activated apoptosis through ROS production and depolarization of the mitochondrial membrane [73]. 

Regarding clinical evidence, a small retrospective study reported that the association of itraconazole with conventional chemotherapy (docetaxel, gemcitabine and carboplatin) had a positive effect in the overall survival and induced a partial response in some of the patients with refractory metastatic pancreatic cancer [127]. In addition, a case report study attributed to itraconazole the reduction of tumor size in a patient with unresectable pancreatic cancer, which allowed the patient to undergo curative surgery [128]. 

#### 3.1.9. Parbendazole

This anthelmintic drug that is only approved for veterinary use, was also studied as a drug-repurposing candidate for the treatment of PDAC as a single agent or in combination therapy. Parbendazole decreased the cell viability and impaired the cell proliferation, clonogenicity and migration in different PDAC cell lines [74]. It also induced apoptosis, DNA damage, promoted G2/M cell cycle arrest and affected the tubulin distribution [74]. Moreover, when combined with gemcitabine, parbendazole decreased the cell viability in a dose-dependent manner, suggesting a chemosensitizing effect for this anthelmintic drug in PDAC cells [74].

#### 3.1.10. Verteporfin and Protoporphyrin IX

Verteporfin is a photosensitizer agent that is used in photodynamic therapy for the treatment of aged-related macular degeneration by the generation of singlet oxygen, which induces cell death. Several studies have focused on the effect of verteporfin-based photodynamic therapy in the treatment of pancreatic cancer [129,130,131]. Regarding protoporphyrin IX, this drug is used in photodynamic therapy in glioma, due to its tumor-localizing properties [132]. However, it appears that these drugs by themselves, without light excitation, may also have antitumor effects [75,133]. Indeed, a study conducted by Acedo et al. (2019) demonstrated that verteporfin and protoporphyrin IX inhibited PDAC cell proliferation and induced apoptosis through the activation of tumor suppressor TAp73 and its proapoptotic targets PUMA, Bax and Bid, without any effect in a nontumoral cell line [75]. Moreover, these drugs induced the production of ROS species in vitro, most likely through the inhibition of thioredoxin reductase, a key redox regulator [75]. In another study, verteporfin partially reversed the effects of Platelet-Derived Growth Factor (PDGF)-BB on cell proliferation, anoikis resistance and cell migration in vitro by suppressing the Hippo/YAP signaling pathway [76]. The Hippo tumor suppressor pathway consists of a serine kinase cascade that regulates cells survival and tissue growth, partly by blocking the Yes-associated protein (YAP) and Tafazzin (TAZ) [134]. YAP is responsible for the expression of several proto-oncogenes (e.g., CTGF, KRAS and Wnt/β-catenin) and acts as a crucial transcription factor in promoting tumor formation and development [134,135]. Interestingly, YAP is also a downstream target of the KRAS signaling, and the increased expression of the YAP signaling network is correlated with poorer survival in PDAC patients [136].

#### 3.1.11. Olanzapine, Penfluridol, Pimozide and Trifluoperazine

A lower incidence of some types of cancer was found in patients treated for schizophrenia [137]. Moreover, antipsychotics of the diphenylbutylpiperidine class, like penfluridol and pimozide, are known calcium channel antagonists and can bind to D2 dopamine receptor-binding sites, which is upregulated in many cancers and intimately related to stemness [138,139].

Chien et al. (2015) demonstrated that penfluridol suppressed the cell proliferation of several PDAC cell lines without affecting the normal pancreatic epithelial cell line and, also, sensitized PDAC cells to gemcitabine treatment [77]. Moreover, penfluridol promoted apoptosis and cell cycle arrest by targeting the proteins phosphatase 2A (PP2A), SRC, AKT and p70S6k, which are key players in pancreatic tumorigenesis [77]. These results were in agreement with the ones observed by Ranjan et al. (2016), who showed that, in vitro, penfluridol induced apoptosis and blocked PDAC cell growth via autophagy and, in vivo, impaired tumor growth in different PDAC models [78]. In a subsequent study, the last authors proposed that the penfluridol-induced autophagy might also be linked to stress of the endoplasmic reticulum (ER) through the upregulation of ER stress markers, such as BIP, CHOP and IRE1a [79]. 

Pimozide is another antipsychotic candidate that has been investigated for the treatment of pancreatic cancer. Jandaghi et al. (2016) found that this drug reduced the growth and migration of PDAC cells through the inhibition of the dopamine receptor D2 (DRD2) protein. Moreover, a DRD2 blockage led to ER stress in pancreatic tumor cells, inducing cell cycle arrest, apoptosis and activation of the Unfolded Protein Response (UPR) [80]. Similar mechanisms were observed by Huang et al. (2019) for trifluoperazine, an antipsychotic drug from the phenothiazines family. PDAC cells treated with trifluoperazine showed a significant impairment in mitochondrial and ER homeostasis, prompting apoptosis and necroptosis. In addition, these authors verified that trifluoperazine-treated cells activated the ubiquitin-proteasome system (UPS) as a compensatory mechanism, and when PDAC-derived primary cells were cotreated with a proteosome inhibitor, the sensitivity to trifluoperazine increased up to 10 times [81].

Another antipsychotic that was also investigated as a drug-repurposing candidate in pancreatic cancer is olanzapine, which is considered an atypical antipsychotic drug due to its nonexisting or few adverse side effects. Besides its antipsychotic activity, olanzapine has an antiemetic effect, also being used in the prevention and treatment of chemotherapy-induced nausea and vomiting. In vitro studies demonstrated that olanzapine suppressed the expression of the antiapoptotic protein survivin in CSC lines established from PDAC cell lines and sensitized cancer cells to chemotherapeutics agents, such as 5-FU, gemcitabine and cisplatin, without causing any toxicity to normal cells such as fibroblasts [82]. 

#### 3.1.12. Disulfiram

The anticancer activity of disulfiram (DSF), which is used in the treatment of chronic alcoholism, has been demonstrated in numerous in vitro and in vivo studies for different types of cancer. Moreover, its well-established chemo- and radio-sensitizing effect makes disulfiram a promising candidate for drug repurposing [140]. Due to its ability to chelate copper (Cu), disulfiram forms DSF-Cu^2+^ complexes that block the 26S proteasome activity and interfere with NF-kB, a transcription factor that controls cell proliferation and survival [141]. Disulfiram/Cu induced ER stress through activation of the IRE1a-XBP1 pathway in PDAC cell lines, activating autophagy-dependent apoptosis [83]. Moreover, another study reported that disulfiram/Cu targeted both PDAC stem and non-stem cells when combined with chemotherapy or chemoradiation [84]. In vivo, it was demonstrated that the combination treatment consisting of DSF/Cu + 5-FU + radiotherapy was the most effective in inhibiting tumor growth, when compared to 5-FU + radiotherapy or to FOLFIRINOX + radiotherapy [84]. In this study, disulfiram/Cu was also shown to inhibit the NF-kB pathway and to downregulate the stemness-related genes, such as HER2, c-myc and SOX9 [84]. 

#### 3.1.13. Bazedoxifene

The selective estrogen modulator bazedoxifene is commonly used for osteoporosis prevention and treatment; however, due to its activity as a STAT3 inhibitor, increasing attention has been drawn to this molecule in the oncological field. In vitro and in vivo results demonstrated that bazedoxifene impaired the growth of pancreatic cancer cells with a persistent STAT3 activation. Additionally, in combination with paclitaxel or gemcitabine, bazedoxifene acted synergistically and inhibited the cell viability, as well as cell migration, in pancreatic cancer cells [85].

#### 3.1.14. Ibrutinib

Massó-Vallés et al. (2015) reported that the drug ibrutinib, which is approved for the treatment of mast cells lymphoma as a suppressor of Bruton’s tyrosine kinase, could impair PDAC cell growth and reduce tumor fibrosis in in vivo models. Moreover, ibrutinib improved the survival and response to gemcitabine in a transgenic mouse model of PDAC [86]. Importantly, these authors observed that mast cells play a major role in collagen deposition and that the antifibrotic effect of ibrutinib is mast cell-dependent [86]. Interestingly, other inhibitors of Bruton’s tyrosine kinase have been also studied for the treatment of pancreatic cancer, highlighting its oncogenic role in this cancer [87,88].

#### 3.1.15. Losartan

Although losartan is commonly known as an angiotensin II receptor antagonist, to treat high blood pressure, it also displays antifibrotic properties. A study conducted by Diop-Frimpong et al. (2011) found that this drug inhibited collagen I synthesis in PDAC tumors and enhanced the transport and distribution of injected pegylated liposomal doxorubicin (Doxil) in orthotopic pancreatic tumors. Although losartan by itself did not impact tumor growth, a combination with Doxil decreased the tumor size by 50% in comparison with the Doxil treatment alone [89]. These results highlight the potential of losartan as an adjuvant drug with chemotherapy, which has been evaluated in several clinical trials (see Table 3).

#### 3.1.16. Pentoxifylline and Pirfenidone

Kim et al. (2017) demonstrated that the vasodilator pentoxifylline increased the efficacy of gemcitabine in vivo by improving the drug delivery, which led to a 50% decrease in tumor growth. Reductions in the levels of collagen I and in the number of activated fibroblasts were observed but not in vessel density, suggesting that pentoxifylline also has an antifibrotic effect [90]. 

The antifibrotic activity of pirfenidone, which is used to treat idiopathic pulmonary fibrosis, has also been studied in pancreatic cancer. Kozono et al. (2013) reported that pirfenidone inhibited the in vitro proliferation, invasiveness, and migration of PSCs, which are intricately connected to desmoplasia in PDAC and contribute to the malignancy and chemoresistance of cancer cells. Pirfenidone also reduced the expression of genes associated with tumor–stromal interactions in human PSCs from PDAC patient samples and in vivo, it inhibited the growth of PSCs and presented synergistic effects with gemcitabine [92]. Another work also reported that pirfenidone may directly target PDAC cells, in addition to fibroblasts and stromal cells [93].

Xavier et al. (2021) also demonstrated that the treatment of PDAC tumor cells with pentoxifylline and pirfenidone could partially reverse the chemoresistance to gemcitabine. These drugs are inhibitors of chitinase 3-like-1 and fibronectin, respectively, two proteins that the authors found to be in the cargo of extracellular vesicles shed by human macrophages and are responsible of gemcitabine resistance in vitro [91].

## 4. Repurposed Drugs for Pancreatic Cancer in Clinical Trials 

Given all the advantages of drug repurposing, there are several drugs previous approved for other diseases that are currently under different phases of clinical trials for the treatment of pancreatic cancer. Based on the database “clinicaltrials.gov”, this review summarizes the most relevant classes of drugs under investigation in clinical trials for the treatment of pancreatic cancer (Table 3). 

### 4.1. Antidiabetics

In general, patients with diabetes are more prone to several types of cancer that may be related with a chronic increase in glycemia, partially contributing to tumor development [3]. In fact, around 80% of pancreatic cancer patients have concomitant diabetes, which is regarded as a poor prognostic factor. On the other hand, damaged pancreatic tissue promotes a replacement of islet and beta cells by malignancy, leading to diabetes. Consequently, diabetes is both a potential cause and effect of this disease [98]. Thus, the possibility of repurposing metformin as an adjuvant drug in chemotherapy for pancreatic cancer treatment has emerged. Indeed, several phase II clinical trials, in which metformin was added to the traditional chemotherapy regimens compared with chemotherapy given alone, are being performed on pancreatic cancer (NCT01210911, NCT01666730, NCT01167738, NCT02005419 and NCT01971034) [142,143,144,145,146]. So far, only two of these studies have had their results published (NCT01210911 and NCT01971034). The study NCT01210911 showed that adding metformin in a conventional antidiabetic dosage to gemcitabine and erlotinib treatments in patients with advanced or metastatic pancreatic cancer did not improve the outcomes in patients. Although not encouraging, these results suggested that, for future trials, the focus should be on patients with hyperinsulinemia or patients with tumors-expressing markers of sensitivity to energetic stress, such as the loss of function of the AMP kinase, a key regulator of cellular energy homoeostasis [101]. Unfortunately, regarding the clinical trial NCT01971034, the authors concluded that the addition of metformin to paclitaxel in patients with locally advanced or metastatic pancreatic cancer whose disease progressed when treated with gemcitabine did not improve the overall survival or progression-free survival [147].

Another antidiabetic drug studied with promising potential in the treatment of pancreatic cancer is pioglitazone, a Proliferator-activated receptor-γ (PPAR-γ) ligand, approved by the FDA for type 2 diabetes [148]. PPAR-γ is a nuclear receptor that functions as a transcription factor in different tissues. PPAR-γ ligands, such as pioglitazone, are being studied for their ability to inhibit numerous cancer cell processes [149]. In a recently closed phase II clinical trial (NCT01838317), pioglitazone was added to the standard chemotherapy, aiming to evaluate its effects on glucose and insulin metabolism, tumor size, weight gain and the general quality of life of pancreatic cancer patients. The results of this study are not published yet [150].

### 4.2. Vitamins

Ascorbic acid is a water-soluble vitamin, commonly named vitamin C, which appears abundantly in fresh fruit, especially blackcurrants, citrus fruit and strawberries, and in most fresh vegetables [151]. Many health benefits have been associated with this vitamin, such as antioxidant, antiatherogenic, anticarcinogenic and immunomodulatory [152]. Curiously, pharmacological concentrations of ascorbate can generate hydrogen peroxide, inducing oxidative damage, with this cytotoxicity apparently being selective to different types of cells (with a higher toxicity to cancer cells than to normal cells). Thus, several authors became interested in the clinical repurposing of intravenous high doses of ascorbate in pancreatic cancer. The doses reported in different studies vary. However, several trials used around 25 to 100 g per infusion [153].

In a phase II clinical trial of the study NCT01905150, the authors compared the administration of G-FLIP (low doses of gemcitabine, fluorouracil, leucovorin, irinotecan and oxaliplatin) and G-FLIP-DM (low doses of gemcitabine, fluorouracil, leucovorin, irinotecan, oxaliplatin, docetaxel and mitomycin C) alone versus their combinations with a high dose of ascorbic acid in patients with advanced pancreatic cancer [154]. The results of this study demonstrated that the conventional therapy combined with high doses of ascorbic acid caused an overall survival at 11 months of 75% and a disease control rate (including complete response, partial response and stable disease) of 73%. Regarding the toxicity profile, the hematologic adverse events registered were mostly mild (Grade 1 and 2), some Grade 3 and no Grade 4. The nonhematologic adverse events were also mostly Grade 1 or 2, except 6% of Grade 3 with diarrhea and thromboembolism. Furthermore, no Grade 3 or 4 nausea, vomiting or neuropathy were registered. Therefore, the combination was considered well-tolerated by the patients and should be further explored, especially in elderly patients and patients who are not candidates for high doses of conventional therapy [155]. Currently, there are several ongoing clinical trials that aim to explore the potential use of this vitamin in pancreatic cancer using different approaches: high doses of vitamin C alone (NCT03146962 and NCT02905578), in combination with metformin (NCT04033107), in combination with radiation therapy (NCT03541486) or in combination with standard chemotherapy regimens (NCT03410030). So far, no results from these trials have been published [156,157,158,159,160].

Paricalcitol is a synthetic analog of calcitriol, the metabolically active form of vitamin D, approved by the FDA for the prevention and treatment of secondary hyperparathyroidism associated with chronic kidney disease [161]. Interestingly, studies have shown an association between vitamin D and cancer due to 1α,25(OH)2D, the biologically active form of vitamin D, which functions as a hormone with several activities, including the inhibition of cellular proliferation, induction of differentiation and apoptosis. However, the systemic administration of 1α,25(OH)2D3 leads to lethal hypercalcemia, not allowing its use as a possible cancer treatment. To overcome this issue, synthetic analogs with less calcemic effects and more potent growth inhibitors have been developed, including Paricalcitol [162]. This vitamin D analog has been included in several phase II clinical trials (NCT04617067, NCT04524702, NCT03520790, NCT04054362, NCT03138720 and NCT03415854) to study its effect in combination with the usual chemotherapy regimens in patients with pancreatic cancer [163,164,165,166,167,168]. These studies are still ongoing, and thus, no results are so far available.

### 4.3. Hydroxychloroquine and Chloroquine

Hydroxychloroquine and chloroquine are 4-aminoquinoline compounds. While hydroxychloroquine is approved by the FDA for the treatment of malaria, lupus erythematosus and rheumatoid arthritis, chloroquine is approved for the treatment of malaria and extraintestinal amebiasis [169,170]. These compounds may have a role in cancer treatment due to their ability to inhibit autophagy, which enhances the efficacy of several chemotherapeutic and targeted therapies [171]. Thus, repurposing these drugs may be extremely promising for autophagy-dependent malignant tumors, including pancreatic cancer, known to be highly dependent on this mechanism for survival [3,172,173]. Therefore, there are several ongoing clinical trials aiming to evaluate the combination of hydroxychloroquine with the standard chemotherapy regimens and/or radiation (NCT04524702, NCT04669197, NCT01506973 and NCT01494155) [164,174,175,176]. Chloroquine has also been tested in a phase I trial (NCT01777477) to evaluate its safety and preliminary efficacy when combined with gemcitabine [177]. The results have shown that this combination was well-tolerated in patients with metastatic or unresectable pancreatic cancer [178] and, therefore, should be further explored.

### 4.4. Tyrosine Kinase Inhibitors (TKIs)

Currently, there are two TKIs approved for the treatment of pancreatic cancer—namely, erlotinib and sunitinib [179]. These drugs were previously approved by FDA for other types of cancers, such as metastatic non-small cell lung cancer and gastrointestinal or advanced renal cell carcinoma, respectively [180,181], making them the first successful examples of TKIs approved for pancreatic cancer.

Nowadays, other similar molecules are under clinical trials, such as ruxolitinib, which is a selective inhibitor of the Janus kinase 1 (JAK1) and Janus kinase 2 (JAK2) enzymes, approved by the FDA for the treatment of post-essential thrombocythemia myelofibrosis, post-polycythemia vera myelofibrosis, high- and intermediate-risk myelofibrosis and refractory polycythemia vera [182]. Studies have shown that the JAK/signal transducer is at least partly involved in the systemic inflammatory response showed by patients with pancreatic cancer. In a phase II clinical trial (NCT01423604) with metastatic pancreatic cancer patients resistant to gemcitabine, those receiving ruxolitinib plus capecitabine had a survival improvement, especially the patients showing systemic inflammation, when compared with the ones receiving the placebo plus capecitabin [183,184]. Due to this promising data, two clinical trials in phase III were conducted (NCT02117479 and NCT02119663) to evaluate the effects of ruxolitinib in combination with capecitabine in patients with advanced/metastatic pancreatic cancer after disease progression or who presented an intolerance to the first-line therapy. Unfortunately, both trials were terminated earlier due to an interim futility analysis (it appears that the experimental arm was unlikely to show better results than the control arm if the trial was continued to the final analysis). Although well-tolerated, the efficacy of ruxolitinib in improving patients’ survival and clinical outcomes has not yet been proven [185,186,187].

Another example of a TKI is masitinib, which is approved by the European Committee for Medicinal Products for Veterinary Use for the treatment of mast-cell tumors with a mutation in the receptor protein c-kit in dogs [188]. This drug was evaluated in a phase III clinical trial (NCT00789633) in combination with gemcitabine in pancreatic cancer patients, revealing an improvement in the overall survival when compared with gemcitabine alone [189,190]. Later, similar studies were conducted in another phase III clinical trial (NCT03766295), showing that masitinib was able to improve the survival and reduce the pain in patients with unresectable locally advanced pancreatic cancer [191,192].

Sorafenib is another potent TKI already approved by the FDA to treat unresectable hepatocellular carcinoma, advanced renal cell carcinoma and thyroid carcinoma [193]. This drug interferes with Raf-1, a member of the RAF/MEK/ERK signaling pathway, which plays a critical role in pancreatic cancer cell proliferation [194]. Unfortunately, in a phase III trial (NCT00541021) with locally advanced or metastatic pancreatic cancer patients, the administration of gemcitabine with sorafenib failed to demonstrate a superior progression-free survival in comparison with gemcitabine alone [195,196].

### 4.5. Poly (ADP) Ribose Polymerase (PARP) Inhibitors

Poly (ADP) ribose polymerases (PARPs) are a large family of nuclear proteins with an important role in DNA repair pathways, especially in the repair of single-strand breaks [197,198]. PARP inhibitors can prevent DNA repair and allow the accumulation of single-strand breaks. The efficacy of PARP inhibitors is advantageous in the treatment of malignancies that present mutations in homologous recombination repair enzymes, such as breast cancer gene (BRCA), BRCA1 and BRCA2 (being unable to repair these breaks) [199].

The PPAR inhibitor olaparib has previously been approved by the FDA to treat ovarian and breast cancers [200]. Recently, in 2019, the FDA approved olaparib for the treatment of BRCA-mutated metastatic pancreatic cancer. This decision was based on the results from a clinical trial (NCT02184195) that showed a longer progression-free survival when olaparib was added to patient’s treatment regimen [201,202].

Niraparib is another PARP inhibitor that was initially approved for the treatment of fallopian tube cancer, ovarian epithelial cancer and primary peritoneal cancer [203]. Currently, this drug is being investigated in phase II clinical trials (NCT03601923 and NCT03553004) in unresectable or metastatic pancreatic cancer patients possessing germline or somatic mutations in genes involved in DNA repair [204,205].

Another PARP inhibitor that can potentially be repurposed for pancreatic cancer treatment is rucaparib, a drug previously approved by the FDA for the treatment of ovarian and prostate cancer [206]. In a clinical trial (NCT02042378), rucaparib proved to be safe and clinically relevant in advanced pancreatic cancer patients with *BRCA1/2* mutations [207,208]. In another ongoing phase II clinical trial (NCT03140670), rucaparib is being tested on pancreatic cancer patients with a locally advanced or metastatic disease possessing a germline or somatic deletion in *BRCA1/2* or *PALB2* mutations [209].

### 4.6. Monoclonal Antibodies (mAb)

Several clinical trials at different stages are being conducted with monoclonal antibodies (mAb) for the treatment of pancreatic cancer. The most relevant monoclonal antibodies in this context are bevacizumab and cetuximab, since they have already reached phase III clinical trials. Bevacizumab is a vascular endothelial growth factor (VEGF) inhibitor approved by the FDA for the treatment of metastatic colorectal cancer, non-squamous non-small cell lung cancer, glioblastoma, cervical cancer and metastatic renal cell carcinoma [210]. In a phase III clinical trial (NCT01214720), this mAb was tested in combination with gemcitabine and erlotinib in patients with metastatic pancreatic cancer. The results demonstrated that, when bevacizumab was added to gemcitabine and erlotinib treatments, progression-free survival was significantly longer; however, no significant improvement in the overall survival was verified [211,212]. Bevacizumab was also studied in combination with gemcitabine in a phase III trial (NCT00088894) in advanced pancreatic cancer patients. Unfortunately, these authors concluded that bevacizumab did not improved patient survival [213,214]. In spite of the inconsistent data so far, this mAb is still under investigation in at least two phase II clinical trials (NCT03351296 and NCT03193190) [215,216].

Cetuximab is an EGFR antagonist already approved to treat head and neck cancer, as well as colorectal cancer [217]. Several clinical trials have also been conducted in pancreatic cancer. In a phase III trial (NCT00075686), no significant differences in the median survival time and progression-free survival were observed between the combination gemcitabine/cetuximab and gemcitabine alone [218,219].

### 4.7. Other Relevant Examples

Aflibercept is a VEGF inhibitor currently used to treat eye pathologies like neovascular age-related macular degeneration, diabetic macular edema and diabetic retinopathy [220]. A phase III clinical trial (NCT00574275) was conducted to evaluate the effectiveness of aflibercept in increasing the overall survival of metastatic pancreatic cancer patients treated with gemcitabine [221]. Unfortunately, the addition of aflibercept did not bring a survival advantage when comparing with the standard treatment, making the frequency of the adverse events even higher [222].

Celecoxib is a nonsteroidal anti-inflammatory drug approved by the FDA for the treatment of osteoarthritis, rheumatoid arthritis, ankylosing spondylitis, acute pain and primary dysmenorrhea. Celecoxib is a selective Cyclooxygenase-2 (COX-2 inhibitor [223], which is an enzyme known to be upregulated in various gastrointestinal tumors, including pancreatic adenocarcinoma [224]. In phase II clinical trials (NCT00176813 and NCT00068432), celecoxib was combined with standard chemotherapy drugs to determine the overall survival and clinical outcomes of pancreatic metastatic cancer patients [225,226]. Both studies concluded that the administration of celecoxib in combination with gemcitabine is safe and well-tolerated. However, their efficacy was not consistent between the two trials. While, in the clinical trial NCT00068432, the authors concluded that the combination of gemcitabine and celecoxib was beneficial compared to gemcitabine individually, in the NCT00176813 trial, the combination of celecoxib with gemcitabine and cisplatin did not reveal a significant impact on the survival rate and overall survival of the patients [227,228]. In a phase III clinical trial (NCT00486460), celecoxib in combination with gemcitabine and curcumin are being evaluated in advanced or inoperable pancreatic cancer patients [229]. The potential adjuvant anticancer effect of celecoxib is also being evaluated in another clinical trial (NCT01111591) [230]. An ongoing phase II study (NCT03498326) is also evaluating the synergistic effect of celecoxib in combination with gemcitabine for the treatment of resection pancreatic cancer patients [231].

Tolfenamic acid is another nonsteroidal anti-inflammatory drug used to treat migraines that has demonstrated antitumor activity in preclinical pancreatic models, especially when in combination with gemcitabine and radiation therapy. A phase I clinical trial (NCT02159248) was planned, aiming to determine the maximum tolerated dose, safety and the antitumor activity of locally advanced or metastatic pancreatic patients treated with gemcitabine and radiation in combination with tolfenamic acid. Unfortunately, this study was closed prior to enrolling participants, and therefore, no results are presented [232].

Losartan is an angiotensin II receptor blocker approved by the FDA and indicated for the treatment of hypertension and reduction of the risk of stroke in patients with hypertension and left ventricular hypertrophy and diabetic nephropathy [233] (see Section 3.1.15). In a phase II clinical trial (NCT01821729), the authors showed that losartan provides a downstaging of locally advanced pancreatic cancer when combined with a FOLFIRINOX treatment [234,235]. Currently, several ongoing clinical trials at different phases are being conducted to further study the benefit of using losartan in the treatment of pancreatic cancer (NCT04539808 in phase II, NCT03563248 in phase II and NCT04106856 in phase I) [236,237,238].

## 5. Conclusions

**Figure 2:**
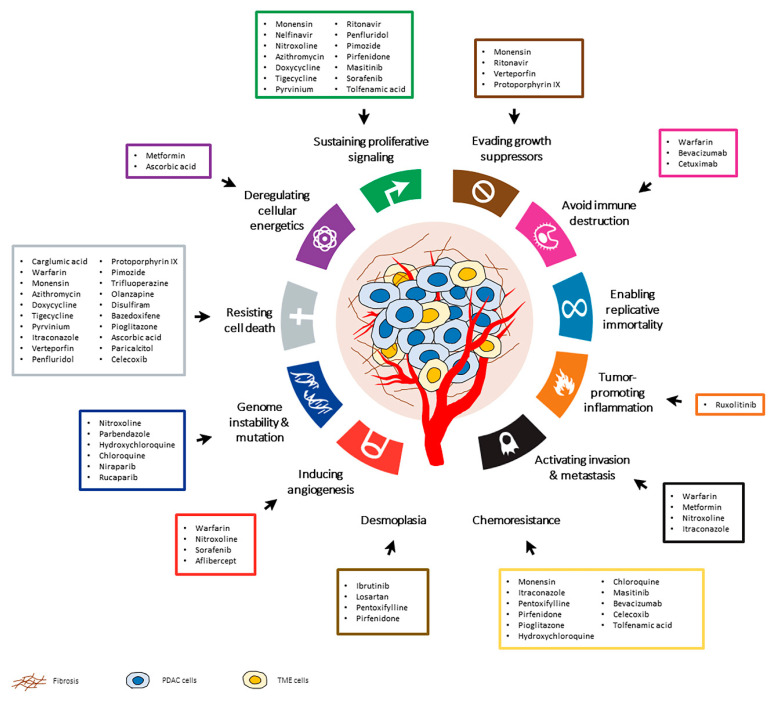
The hallmarks of cancer and other pathological characteristics of pancreatic ductal adenocarcinoma (PDAC) that may be targeted by drug repurposing candidates. These drugs have been investigated in preclinical studies or clinical trials for the treatment of PDAC. Adapted from Hanahan and Douglas et al. 2011.

Drug repurposing is efficient, cost-effective and riskless and no longer a result of serendipity but, rather, a rational process benefiting from systematic approaches and modern technologies (omics, bioinformatics, machine learning, etc.). It is undeniable that this is a resourceful strategy to broaden the therapeutic options in pancreatic cancer and provide a faster response to the main challenges of this disease, particularly to drug toxicity, chemoresistance and desmoplasia. As highlighted in this review, there are numerous drug-repurposing candidates that could potentially be used in PDAC treatments, some of which are already undergoing clinical trials, as summarized in Figure 2. Although additional studies are required to ensure the safety and effectiveness of many of these drug-repurposing candidates, this possibility offers a new hope for pancreatic cancer treatment.

## Figures and Tables

**Table 2:** Selected drug repurposing candidates for treatment of PDAC.

Drug	Pharmacological Class	Original Indication	Evidence			Biological Models	Tested Concentrations	Described Mechanisms	References
			In Vitro	In Vivo	Clinical				
Carglumic acid	Amino acids and derivatives	Hyperammonemia	+	+		Human (AsPC-1 and Capan-1) and Murine (Pan02) pancreatic cancer cell lines Human pancreatic ductal epithelial cell line (HPDE-E6E7) Orthotopic PDAC mouse model	Half maximal inhibitory concentration (IC_50_) = 5–7.5 mM 120 mg/kg once per day (o.d)., 10 days	Induction of apoptosis	[57]
Warfarin	Anticoagulant	Prophylaxis and treatment of venous thrombosis and thromboembolic complication	+	+	+	Human (AsPC-1, Panc-1, Capan-1, Mia PaCa-2 and C5LM2) and murine (Pan02) pancreatic cancer cell lines [58] Syngeneic PDAC mouse model (Pan02) [58] Spontaneous genetic PDAC mouse model (KIC) [58] Human PDAC xenografts mouse models (Panc-1, AsPC-1, Capan-1) [58] KIC model [59] Panc02-SIY mouse model [60]	1.5–3 mM [58] 0.2 mg/kg body weight o.d., 5 days/week [59] 1.25 mg/L [60]	Inhibition of Gas6-induced Axl signaling activated apoptosis and suppressed EMT Immunomodulatory response by inhibition of MerTK signaling	[58,59,60,61]
Metformin	Antidiabetic (Biguanide)	Type 2 diabetes mellitus	+	+	+	Human pancreatic cancer cell lines (BxPC3, Mia PaCa-2 and AsPC-1) [62] KC and KPC mice [63] Human pancreatic cancer cell lines (BxPC3 and Panc-1) [64] KPC mice [64]	250 nM and 1 mM [62] 200 mg/kg o.d. [63] 2 mM [64] 200 mg/kg o.d., 4 weeks [64]	Inhibition of mTOR, STAT3 and TGF-β1/Smad2/3 signaling Activation of AMPK	[62,63,64]
Monensin	Antibiotic (veterinary use)	Ketosis in peri-parturient dairy cow/heifer	+	+		Human pancreatic cancer cell lines (Panc-1 and MiaPaCa-2)	0.5–4 mM	Activation of apoptosis and cell cycle arrest Inhibition of the E2F/DP1, STAT1/2, NF-kB, AP-1 and Elk-1/SRF pathways and suppression of EGFR expression	[65]
Nelfinavir and nitroxoline	Antiviral (Nelfinavir), Antibiotic (Nitroxoline)	Nelfinavir: HIV-1 infection Nitroxoline: Urinary tract infection	+			Human pancreatic cancer cell lines (AsPC-1, Capan-2 and BxPC-3) [66] Human pancreatic cancer cell line (AsPC-1) [67]	Nelfinavir: 3–48 mM [66] Nitroxoline: 16–40 mM [66]; 27 mM [67]	Cell cycle arrest and activation of apoptosis Nitroxoline: ROS production, DNA damage response, mitochondrial depolarization and deregulation of cytosolic iron homeostasis [67]	[66,67]
Azithromycin, doxycycline, tigecycline and pyrvinium	Antibiotic (Doxycycline, Azithromycin, Tigecycline), Anthelmintic (Pyrvinium)	Doxycycline, Azithromycin, Tigecycline: bacterial infections Pyrvinium: gastrointestinal parasitic infections	+	+		Human pancreatic cancer cell line (Panc-1) [68] Human PDAC xenograft mouse model [68] Human pancreatic cancer cell line (MiaPaca-2) [69]	Doxycycline: 10–40 μg/mL [68]; 50µM [69] Azithromycin: 250µM [69] Tigecycline: 50µM [69] Pyrvinium: 250 nM and 500 nM [69]	Induction of apoptosis and cell cycle arrest [68] Impairment of mitochondrial biogenesis and oxidative phosphorylation [69]	[68,69]
Ritonavir	Antiviral	HIV-1 infection	+	+		Human pancreatic cancer cell lines (BxPC-3, MiaPaCa-2 and Panc-1) [70] Genetically engineered PDAC mice models [71]	5–30 µM [70] 125 mg/kg, 28 days [71]	Induction of apoptosis and cell cycle arrest, through Inhibition of E2F-1 and AKT pathway	[70,71]
Itraconazole	Antifungal	Fungal infections	+	+	+	Human pancreatic cancer cell lines (BxPC-3 and Panc-1) [72] KPC mouse model [72] Human pancreatic cancer cell lines (Panc-1, CFPAC-1 and MiaPaCa-2) [73] Human PDAC xenograft mouse model [73]	0.5–10 mM [72] 5–160 mM [73] 40 and 80 mg/kg [73]	Activation of apoptosis Inhibition of TGF-β/SMAD2/3 signaling ROS production and mitochondrial membrane depolarization	[72,73]
Parbendazole	Anthelmintic (veterinary use)	Parasitic infections by nematodes	+			Human pancreatic cancer cell lines (AsPC-1 and Capan-2)	0.06–4 mM	Apoptosis induction, DNA damage, cell cycle arrest and alterations of tubulin distribution	[74]
Verteporfin, protoporphyrin IX	Antineovascularization agent (Verteporfin), Sensitizers in photodynamic therapy (protoporphyrin IX)	Verteporfin: aged-related macular degeneration Protoporphyrin IX: visualization of malignant tissue for malignant glioma	+			Human (Paca-3, MiaPaca-2, Panc-1) and mouse (Panc02) pancreatic cancer cell lines [75] Normal human pancreatic ductal epithelial cell line (HPDE) [75] Human pancreatic cancer cell line (BxPC-3) [76]	2.5 μg/mL [75] 0.1, 0.5 and 1 µM) [76]	Activation of apoptosis via TAp73 activation, Inhibition of thioredoxin reductase Inhibition of Hippo/YAP signaling pathway	[75,76]
Penfluridol	Antipsychotic	Psychological disorders	+	+		Human pancreatic cancer cell lines (MiaPaca-2, Panc-1, SU8686, Panc0504, Panc0403, Panc1005, Panc0203, Panc0327, AsPc1 and BxPc-3) [77] Normal human pancreatic ductal epithelial cell line (HPDE) [77] Human pancreatic cancer cell lines (BxPC-3 and AsPC-1) [78,79]. Orthotopic PDAC tumor model [78,79]	1–60 μM [77] 2.5–10 mM [78,79] 10 mg/kg [78,79]	Apoptosis activation and cell cycle arrest, by targeting of protein phosphatase 2A (PP2A), SRC, AKT and p70S6k ER stress	[77,78,79]
Pimozide	Antipsychotic	Psychological disorders	+			Human pancreatic cancer cell lines (BxPC-3 cells, Panc-1, MiaPaCa-2, Capan-1 and CFPAC-1) [80] Human pancreatic cancer cell line (MiaPaCa-2) [81] PDAC primary cell cultures [81]	0.1–10 mM [80]	Inhibition of DRD2, ER stress, cell cycle arrest, activation of apoptosis and activation of the UPR	[80]
Trifluoperazine	Antipsychotic	Psychological disorders	+	+		Human pancreatic cancer cell line (MiaPaCa-2) PDAC primary cell cultures from xenografts (01008, HN01, JIPC and LIPC) [81]	10–30 mM	Impairment of mitochondrial and ER homeostasis, induction of apoptosis and necroptosis and activation of the UPR	[81]
Olanzapine	Antipsychotic	Psychological disorders	+			Cancer stem cell lines established from human pancreatic cancer cell lines (Panc-1 and PSN-1) [82]	10–100 mM	Inhibition of surviving in CSCs	[82]
Disulfiram	Drugs used in addictive disorders	Treatment of alcohol dependence	+	+		Human pancreatic cancer cell lines (PDAC6 and Panc-1) [83] Human pancreatic cancer cell lines (Panc-1, PDAC-2, PDAC-2 and PDAC-6) [84] Syngeneic PDAC mouse tumor model (Panc02) [84]	0.25 mM [83] 0.2 and 2.5 mM [84] 50 mg/kg/day, 8 days [84]	Activation of autophagy-dependent apoptosis ER stress by activation of the IRE1a-XBP1 pathway Inhibition of the NF-kB signaling pathway and downregulate stemness-related genes (HER2, c-myc and SOX9)	[83,84]
Bazedoxifene	Selective estrogen receptor modulator	Postmenopausal osteoporosis in women at increased risk of fracture	+	+		Human pancreatic cancer cell lines (AsPC-1, PANC-1, HPAF-II, BxPC-3, HPAC and Capan-1) Human PDAC xenograft mouse model	5–20 mM 5 mg/kg/day	Inhibition of STAT3 activation mediated by interleukin 6 (IL-6) and 11 (IL-11)	[85]
Ibrutinib	Antineoplastic agents (protein kinase inhibitors)	Treatment of adult patients with relapsed or refractory mantle cell lymphoma (MCL)		+		Transgenic PDAC mouse model Patient-derived xenograft models	35 mg/kg/day	Mast cell-dependent antifibrotic effect	[86,87,88]
Losartan	Angiotensin II receptor antagonist	Hypertension		+		Orthotopic PDAC mouse model	10–60 mg/kg	Inhibition of collagen I synthesis	[89]
Pentoxifylline	Vasodilator	Patients with chronic occlusive peripheral vascular disorders of the extremities	+	+		Human pancreatic cancer cell line (Capan-1) [90] Human PDAC xenograft mouse model [90] Human pancreatic cancer cell line (BxPC-3 and Panc-1) [91] Human monocytes isolated from blood of healthy donors [91]	50 and 100 mg/kg per day [90] 0.4 mM [91]	Reduction in collagen I and downregulation of alpha-smooth muscle actin and connective tissue growth factor Inhibition of chitinase 3-like-1	[90,91]
Pirfenidone	Antifibrotic	Idiopathic pulmonary fibrosis	+	+		Human PSCs from pancreatic cancer surgical specimens [92] Orthotopic PDAC mouse model [92] Human PDAC xenograft mouse model [92] Human pancreatic cancer cell lines (Panc-1, MiaPaca-2 and BxPC-3) [93] Human skin fibroblasts (ASF-4-1 cells) [93] Human pancreatic cancer cell line (BxPC-3 and Panc-1) [91] Human monocytes isolated from blood of healthy donors [91]	0.1–1 mg/mL [92] 500 mg/kg [92] 0.1–0.5 mg/mL [93] 1 mM [91]	Suppression of desmoplasia through regulation of PSCs Cell cycle arrest and upregulation of p21 of PDAC cells Inhibition of fibronectin	[91,92,93]

EMT, Epithelial-to-Mesenchymal Transition; EGFR, Epidermal Growth Factor Receptor; ROS, Reactive Oxygen Species; ER, Endoplasmic Reticulum; UPR, Unfolded Protein Response; CSCs, Cancer Stem Cells and PSCs, Pancreatic Stellate Cells.

**Table 3:** Summary of the drug-repurposing clinical trials for pancreatic cancer. The information was searched on the database clinicaltrial.gov (https://www.clinicaltrail.gov, accessed between 3 February and 28 February 2021).

Class	Drug	Approved Indications	Trial Identifier	Phase	Title	Results
Antidiabetics	Metformin	Type 2 diabetes mellitus	NCT01210911	II	Metformin Combined With Chemotherapy for Pancreatic Cancer (GEM)	No patient outcome improvement
			NCT01666730	II	Metformin Plus Modified FOLFOX 6 in Metastatic Pancreatic Cancer	Data not available
			NCT01167738	II	Combination Chemotherapy With or Without Metformin Hydrochloride in Treating Patients With Metastatic Pancreatic Cancer (PACT-17)	Data not available. Study closed
			NCT02005419	II	Metformin Combined With Gemcitabine as Adjuvant Therapy for Pancreatic Cancer After Curative Resection	Data not available
			NCT01971034	II	Treatment of Patients With Advanced Pancreatic Cancer After Gemcitabine Failure	No patient outcome improvement
	Pioglitazone		NCT01838317	II	A Phase II Study of Pioglitazone for Patients With Cancer of the Pancreas	Data not available
Vitamins	Ascorbic Acid	Scurvy	NCT01905150	II	Ph 2 Trial of Vitamin C and G-FLIP (Low Doses Gemcitabine, 5FU, Leucovorin, Irinotecan, Oxaliplatin) for Pancreatic Cancer	Favorable toxicity profile
			NCT04033107	II	High Dose Vitamin C Combined With Metformin in the Treatment of Malignant Tumors	Ongoing, recruiting
			NCT03146962	II	High Dose Vitamin C Intravenous Infusion in Patients With Resectable or Metastatic Solid Tumor Malignancies	Ongoing, recruiting
			NCT03541486	II	A Clinical Trial Evaluating the Effect of Pharmacological Ascorbate on Radiation Therapy for Pancreatic Cancer Patients (XACT-PANC-2)	Ongoing, not yet recruiting
			NCT02905578	II	A Phase 2 Trial of High-dose Ascorbate for Pancreatic Cancer (PACMAN 2.1)	Ongoing, recruiting
			NCT03410030	I/II	Trial of Ascorbic Acid (AA) + Nanoparticle Paclitaxel Protein Bound + Cisplatin + Gemcitabine (AA NABPLAGEM) (AA NABPLAGEM)	Ongoing, recruiting
	Paricalcitol	Hyperparathyroidism	NCT04617067	II	Paricalcitol Trial	Ongoing, recruiting
			NCT04524702	II	Paricalcitol and Hydroxychloroquine in Combination With Gemcitabine and Nab-Paclitaxel for the Treatment of Advanced or Metastatic Pancreatic Cancer	Ongoing, recruiting
			NCT03520790	I/II	Paricalcitol Plus Gemcitabine and Nab-paclitaxel in Metastatic Pancreatic Cancer	Ongoing
			NCT04054362	II	Paricalcitol Addition to Chemotherapy in Patients With Previously Untreated Metastatic Pancreatic Ductal Adenocarcinoma (PINBALL)	Ongoing, recruiting
			NCT03138720	II	Pre-operative Treatment for Patients With Untreated Pancreatic Cancer	Ongoing, recruiting
			NCT03415854	II	Paclitaxel Protein Bound Plus Cisplatin Plus Gemcitabine and Paricalcitol for Pancreatic Adenocarcinoma (NABPLAGEMD) (NABPLAGEMD)	Ongoing
Antimalarials	Hydroxychloroquine	Malaria Lupus erythematosus Rheumatoid arthritis	NCT04524702	II	Paricalcitol and Hydroxychloroquine in Combination With Gemcitabine and Nab-Paclitaxel for the Treatment of Advanced or Metastatic Pancreatic Cancer	Ongoing, recruiting
			NCT04669197	II	Phase II Study of Paclitaxel Protein Bound + Gemcitabine + Cisplatin + Hydrochloroquine as Treatment in Untreated Pancreas Cancer	Ongoing, recruiting
			NCT01506973	I/II	A Phase I/II/Pharmacodynamic Study of Hydroxychloroquine in Combination With Gemcitabine/Abraxane to Inhibit Autophagy in Pancreatic Cancer	Ongoing
			NCT01494155	II	Short Course Radiation Therapy With Proton or Photon Beam Capecitabine and Hydroxychloroquine for Resectable Pancreatic Cancer	Ongoing
	Chloroquine	Malaria Extraintestinal amebiasis	NCT01777477	I	Adjuvant Effect of Chloroquine on Gemcitabine	Combination well tolerated
Tyrosine kinase inhibitors	Ruxolitinib	Myelofibrosis Polycythemia vera	NCT01423604	II	Study of Ruxolitinib in Pancreatic Cancer Patients (RECAP)	Improvement in overall survival
			NCT02117479	III	Study of Ruxolitinib in Pancreatic Cancer Patients (Janus 1)	Well tolerated. No survival improvement
			NCT02119663	III	A Study of Ruxolitinib in Pancreatic Cancer Patients	Well tolerated. No survival improvement
	Masitinib	Mast-cell tumour in dogs	NCT00789633	III	Masitinib in Combination With Gemcitabine for Treatment of Patients With Advanced/Metastatic Pancreatic Cancer	Improvement in overall survival
			NCT03766295	III	Masitinib Plus Gemcitabine in Pancreatic Cancer	Improvement in survival and pain reduction
	Sorafenib	Hepatocellular carcinoma Renal cell carcinoma Thyroid carcinoma	NCT00541021	III	Gemcitabine With or Without Sorafenib in Treating Patients With Locally Advanced or Metastatic Pancreatic Cancer	No improvement in free survival
Poly (ADP-ribose) polymerase inhibitors	Niraparib	Fallopian tube cancerOvarian epithelial cancer Primary peritoneal cancer	NCT03601923	II	Niraparib in Patients With Pancreatic Cancer	Ongoing, recruiting
			NCT03553004	II	Niraparib in Metastatic Pancreatic Cancer After Previous Chemotherapy (NIRA-PANC): a Phase 2 Trial (NIRA-PANC)	Ongoing, recruiting
	Rucaparib	Ovarian cancer Prostate cancer	NCT02042378	II	A Study of Rucaparib in Patients With Pancreatic Cancer and a Known Deleterious breast cancer gene (BRCA) Mutation	Safe and clinically relevant
			NCT03140670	II	Maintenance Rucaparib in BRCA1, BRCA2 or PALB2 Mutated Pancreatic Cancer That Has Not Progressed on Platinum-based Therapy	Ongoing
Monoclonal Antibodies	Bevacizumab	Colorectal cancerNon-small cell lung cancer Glioblastoma Cervical cancer Renal cell carcinoma	NCT01214720	III	A Study of Avastin (Bevacizumab) Added to a Chemotherapeutic Regimen in Patients With Metastatic Pancreatic Cancer	Improvement in progression-free survival No improvement in overall survival
			NCT00894	III	Gemcitabine With or Without Bevacizumab in Treating Patients With Locally Advanced or Metastatic Pancreatic Cancer	No improvement in overall survival
			NCT03351296	II	Two Chemotherapy Regimens Plus or Minus Bevacizumab (BETTER 2)	Ongoing, recruiting
			NCT03193190	I/II	A Study of Multiple Immunotherapy-Based Treatment Combinations in Participants With Metastatic Pancreatic Ductal Adenocarcinoma (Morpheus-Pancreatic Cancer)	Ongoing, recruiting
	Cetuximab	Head and neck cancer Colorectal cancer	NCT00075686	III	S0205 Gemcitabine w/or w/o Cetuximab as First-Line Therapy in Locally Advanced Pancreas Cancer	No improvement in median survival time or progression-free survival
Vascular Endothelial Growth Factor Inhibitors	Aflibercept	Macular degeneration Diabetic macular edema Diabetic retinopathy	NCT00574275	III	Aflibercept Compared to Placebo in Term of Efficacy in Patients Treated With Gemcitabine for Metastatic Pancreatic Cancer (VANILLA)	No improvement in overall survival; high frequency of adverse effects
Nonsteroidal Anti-Inflammatories	Celecoxib	Osteoarthritis Rheumatoid arthritis Ankylosing spondylitis Acute pain Dysmenorrhea	NCT00176813	II	Gemcitabine, Cisplatin, and Celecoxib Treatment of Metastatic Pancreatic Cancer	Safe and well tolerated. No improvement in survival rate or overall survival
			NCT00068432	II	Gemcitabine and Celecoxib in Treating Patients With Metastatic Pancreatic Cancer	Safe and well tolerated. Improvement in overall survival
			NCT00486460	III	Phase III Trial of Gemcitabine, Curcumin and Celebrex in Patients With Advance or Inoperable Pancreatic Cancer	Ongoing
			NCT01111591	IV	Cyclooxygenase-2 Inhibitor for Adjuvant Anticancer Effect in Patients With Biliary-pancreas Cancer	Ongoing
			NCT03498326	II	Gemcitabine and Celecoxib Combination Therapy in Treating Patients With R0 Resection Pancreatic Cancer (GCRP)	Ongoing, recruiting
	Tolfenamic Acid	Migraine	NCT02159248	I	Tolfenamic Acid, Gemcitabine and Radiation for Locally Advanced or Metastatic Pancreatic Cancer Requiring Radiation	Withdrawn
Angiotensin II receptor blockers	Losartan	Hypertension	NCT01821729	II	Proton w/FOLFIRINOX-Losartan for Pancreatic Cancer	Reduction in the locally advanced pancreatic cancer
			NCT04539808	II	NeoOPTIMIZE: Early Switching of mFOLFIRINOX or Gemcitabine/Nab-Paclitaxel Before Surgery for the Treatment of Resectable or Borderline Resectable Pancreatic Cancer	Ongoing. Not yet recruiting
			NCT03563248	II	Losartan and Nivolumab in Combination With FOLFIRINOX and SBRT in Localized Pancreatic Cancer	Ongoing, recruiting
			NCT04106856	I	Losartan and Hypofractionated Rx After Chemo for Tx of Borderline Resectable or Locally Advanced Unresectable Pancreatic Cancer (SHAPER)	Ongoing, recruiting
